# A large-scale physiological model of Inferior Olive neurons reveals climbing fiber intra-burst frequency depends on Olivocerebellar axon morphology

**DOI:** 10.1186/1471-2202-15-S1-P149

**Published:** 2014-07-21

**Authors:** James Kozloski, John Wagner, Heraldo Memelli, Viatcheslav Gurev

**Affiliations:** 1 Computational Biology Center, IBM T.J. Watson Research Center, NY, USA; 2 IBM Research Collaboratory for Life Sciences-Melbourne, Carlton, Australia; 3 State University of New York at Stony Brook, NY, USA

## 

We have constructed a large-scale structural model of the Inferior Olive. Using this model as input to our neural tissue simulator, we constrained a multi-compartment (>1000 compartments) model of an olivary neuron, including dendrites, a cell body, and axon. Our physiological model derives from Schweighofer et al. [1], with changes to its several channel conductances (Na, K, Ca_h_, Ca_l_, K_Ca_, and h) targeted to neuronal branches in order to replicate olivary oscillations in our more detailed structure, which includes a full-scale olivocerebellar axon and climbing fibers. Our implementation also computes detailed Ca^2+^ balance solved in parallel with membrane potential and channel currents for each compartment. Furthermore, a larger circuit model comprising these neurons includes glomeruli subject to anatomical constraints on neuronal touch detection, and targets to them a collection of gap junctions, which contribute to both Ca^2+^ balance and electrotonic coupling.

**Figure 1:**
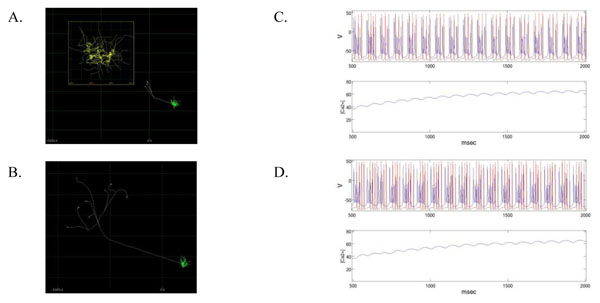
**A.** Short axon (grey) Olivo-cerebellar neuron. Full dendritic morphology (inset) is downsampled to create dendritic morphology (green). **B.** Identical dendrites with long axon. **C.** Short axon bursts. **D.** Long axon bursts.

Previous work has demonstrated that spike generation and bursting properties of olivary neurons depend on axonal lengths [2], but these lengths varied only from 0-300 μm in the slice preparation studied. Our structural model, inspired by measures of real Olivocerebellar axons [3], employs a 3-D mesh of the Purkinje cell layer of rat cerebellum to constrain axonal morphology and length (Figure 1A, B). This method generates a distribution of axonal lengths that more closely resembles the *in vivo* distribution, which is on the order of millimeters.

With our simulator, we explored how preserving precise dendritic morphology, channel distributions, and gap junctional coupling, while varying axonal lengths according to our distribution, affects model dynamics. We demonstrate that the intra-burst spike frequency and number depend on these variations in axonal lengths from 1-5 mm (Figure 1C, D). Therefore, we argue that axons should not be considered structural constraints on only microcircuit connectivity in neural tissue simulation, but also on the dynamics of neuronal spike generating systems.
